# Content-based image retrieval of Indian traditional textile motifs using deep feature fusion

**DOI:** 10.1038/s41598-024-56465-9

**Published:** 2024-05-01

**Authors:** Seema Varshney, Sarika Singh, C. Vasantha Lakshmi, C. Patvardhan

**Affiliations:** 1 Department of Physics and Computer Science, Dayalbagh Educational Institute, Agra, 252008 India; 2 Department of Electrical Engineering, Dayalbagh Educational Institute, Agra, 252008 India

**Keywords:** Neuroscience, Engineering

## Abstract

In the fast-paced fashion world, unique designs are like early birds, grabbing attention as online shopping surges. Fabric texture plays an immense role in selecting the perfect design. Indian Traditional textile motifs are pivotal, showing rich cultural origins and attracting worldwide art fanatics. Yet, technology-driven abstract forms are posing a challenge for them. The decline of handmade artistic ability due to computerization is concerning. Crafting new designs associated with the latest trends is time- consuming and requires diligence. In this work an interactive CBIR (content-based image retrieval) system is presented. It utilizes deep features from InceptionV3 and InceptionResNetV2 models to match query designs with a database of traditional Indian textiles. Its performance is tested with Caltech-101, Corel-1K state-of-the-art datasets, and Indian Textiles datasets and the results are shown to be finer than the existing approaches. The similarity-based fine-grained saliency maps (SBFGSM) approach is employed to visualize the importance of features. Our approach combines deep feature fusion with PCA dimensionality reduction and speeds up search using a clustering approach. Relevance feedback is employed to refine the retrievals. This tool is expected to benefit designers by accelerating the design cycles by bridging the gap between human creativity and A.I. assistance.

## Introduction

India has a rich cultural and artistic heritage with diversity in roots. The traditional Indian textile styles are admired worldwide. These include the ”Madhubani Style” from Bihar, “Kalamkari Style” from Andhra Pradesh, “Ajrakh Style” from Gujrat, “Bagh Style” from Madhya Pradesh, “Kashida embroidery style” from Kashmir, “Chikankari embroidery style” from Uttar Pradesh, etc. Innovative new creations are necessary for preserving these diverse art forms as competition has intensified the variety in the market with reduced time to market. However, methodologies adopted in traditional designs suffer from low productivity, causing a substantial time to market. Fashion designers combine traditional designs with their modern ideas to boost the acceptance of the gen-next. Technology intervention is a must to alleviate this problem.

Works done on classifying and identifying textile images using visual features are very few^1^. An instantly searchable database of existent patterns can go a long way in helping designers to produce new designs rapidly. It will also benefit the e-commerce industry and shoppers seeking newer patterns. Hence, it becomes essential to create database systems for retrieving desired textile patterns from image databases accurately and conveniently. Commonly, two types of approaches are used for textile image retrieval. The first is keywords/text-based image retrieval (KBIR). In this approach, designs are manually annotated with keywords reflecting the contents of the images^2^. The keywords are used as keys to build indexes. Users explore for related images by specifying the appropriate keywords. Searching for specific designs is challenging despite- having high retrieval speed because of limited expression ability of keywords. The attributes of traditional Indian art form fabrics are also tough to describe. In fact, keywords are powerless for some details and features that are difficult to describe. Moreover, manual labeling of fabric images is also substantially subjective, leading to uncertainty of retrieval results and the inaccuracy and inefficiency of KBIR. Manual annotation is expensive and time-consuming, limiting the efficacy of such attempts. These shortcomings in KBIR led to the advancement of retrieval approaches based on content, dubbed content-based image retrieval (CBIR), the second approach for textile image retrieval.

Compared with KBIR, CBIR is more objective^3–5^ and uses image content to retrieve images to avoid the influence of human subjectivity on the result. It has gathered the attention of researchers across several disciplines, like fabric and fashion design^6^, art galleries, remote sensing, and medical imaging. The first commercial version of the CBIR system was created by I.B.M., named query by image content (QBIC)^7^. This system utilizes a combination of color, shape, and texture. It allows users to query using user-constructed drawings, sketches, and example images.

The CBIR process calculates a feature vector that characterizes image properties, for example color, shape, and texture and is saved in the image features database. These feature vectors describe the images’ structural and spatial properties, which are used to retrieve similar images from the image database^8,9^. When a user provides a query image in a CBIR system, it generates a feature vector of that image and compares it with the images’ feature vectors in the datasets. The similarity comparison is made using some distance metric, and the permissible or minimum distances are employed to determine similar or matched images. Different distance measures^10^ have been utilized, such as Jeffrey divergence, Kullback–Leibler divergence, Minkowski-form distance, and histogram intersection. Euclidean distance is the most popular as it is simple and involves low-cost computation. Traditional CBIR techniques use low-level features like color, shape, and texture to represent images and retrieve admissible images to the query image from the image database. This type of retrieval is helpful in small and specialized domains. However, the low-level features approach might return different results if a user tries to obtain images with the same object in the foreground but with different backgrounds.

The critical hurdle faced in the content-based image retrieval (CBIR) domain is the semantic gap between the low-level visual characteristics extracted from images and the corresponding high-level human perceptions. This gap hinders the effective retrieval of images in reference of their content and the meaningful interpretation desired by users. With the latest research advancements in deep-learning neural networks, the outcomes of CBIR systems have gotten a boost. These deep models enable us to handle the semantic gap by extracting both higher-level and low-level features from an image. The efficacy of methods rooted in convolutional neural networks (CNNs) has been empirically demonstrated. Krizhevsky et al.^11^ recommended a method for retrieval of image, which revolved around a seven-layer CNN and demonstrated good performance on ImageNet^12,13^. Babenko et al.^14^ recommended a method for compressing and reducing the features of CNN using P.C.A. and achieved good performance. Enthusiast have obtained new approaches like classification-based CBIR^15^, which uses machine learning to lower the semantic gap and maximize retrieval accuracies. CBIR systems also use relevance feedback, where the user can progressively refine the search results by marking images in the outputs as relevant or non-relevant (or a range of values) to bridge the gap between the high-level semantic concepts in the user’s mind and low-level image features^16^.

This paper proposes an interactive CBIR system for traditional Indian art forms. The idea is to decrease the time of creating new designs to meet the ever-growing demand for fabric designs in the market. The main challenge in this endeavor was the non-availability of a ready database of different Indian traditional art forms. Therefore, one was created from scratch in our previous work^17,18^. However, it was a small dataset. We have extended the same in this work. Here, an explicable user interactive CBIR is proposed using the fusion of deep features by selecting some pre-trained CNN models trained for significant image classification problems. An individual pre-trained CNN (learner) may fall short of expectations due to constraints in response space, misalignment in hypothesis space, or getting trapped in local minimums. Feature fusion technique is proposed to mitigate this issue and provide improved results. Features visualization is proposed by the similarity-based fine-grained saliency maps (SBFGSM) approach to display the significance of fusion features compared to single model features. Moreover, classification in the dataset has been established to reduce retrieval time, and it works faster without sacrificing overall retrieval performance. The resulting approach outperforms CBIR methods in terms of retrieval accuracy. The approach is relatively fast. We extend this approach by incorporating user feedback (“Relevance Feedback”) in the loop, further improving retrieval.

The remaining paper is organized as below. “Literature survey” provides a literature survey. “Methodology” highlights the driving force behind this approach, reviews the key ingredients, and explores the attributes of the derived features from pre-trained CNNs. The dataset description, similarity measures, and performance evaluation are discussed in “Details of the proposed algorithm and performance evaluation”. “Results” presents the results of extensive experiments. “Simulated visualization using similarity-based fine-grained saliency maps” presents the features visualization approach. “Quick response CBIR system” discusses the time complexity and retrieval efficiency of the recommended approaches. We conclude our work in “Conclusion”.

## Literature survey

Recent endeavors in fabric pattern image retrieval are largely divided into (1) feature extraction using handcrafted feature-based methods and (2) feature extraction using automatic learning-based methods.

### Feature extraction using handcrafted methods

Handcrafted methods commonly adopt pixel-level descriptors, such as MPEG-7, image color histogram, histogram of oriented gradient (HoG) descriptor, color moment (CM), scale-invariant feature transform (SIFT) key point descriptor, Gabor, grey level co-occurrence matrices (GLCM), and local binary pattern (LBP) to fabric images. These methods heavily depend on feature engineering. Arora et al.^19^ uses a support vector machine classifier for retrieving textile images, and Xiang et al.^20^utilizes a non-subsampled contourlet transform (NSCT) feature descriptor using a relevance feedback approach for patterned fabric image retrieval.

However, most of the researchers use a blend of two or more feature descriptors to represent fabric images and attain better retrieval accuracy than individual ones^21–27^. These methods are limited to small datasets. Slight jitters in scale or details significantly affect the retrieval results and demonstrate the necessity for more robustness in these methods. Color features are susceptible to illumination, while shape and texture features are susceptible to geometrical shifts. This is the reason that high-level features are also needed, and low-level features like pixel values and others are not enough.

### Feature extraction using automatic learning-based methods

**Figure 1:**
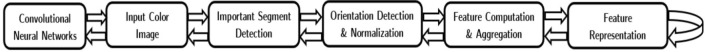
CNN-based feature representation pipeline.

In the last decade, a shift has been observed in feature representation from hand-engineering to deep learning. Deep learning is a hierarchical feature representation technique to learn abstract features from data, that are essential for the dataset and application at hand. This section discusses automatic feature learning-based methods. The CNN feature representation pipeline is depicted in Fig. 1. CNNs require large amounts of data. Therefore, training it on large datasets provides the requisite knowledge base to identify objects. A deep learning network performed outstanding retrieval in the ImageNet challenge^13^. The basic CNN model motivated other deep learning-based approaches, such as AlexNet, VGGNet, GoogleLeNet, Microsoft ResNet, etc., in the image retrieval domain.

Previous studies^28,29^ have trained CNN models for image retrieval systems of wool fabric using classified search, demonstrating the ability of CNNs to learn binary codes and features from labeled data. Whereas Sun et al.^30^ integrate CNNs and hash encoding to reduce feature dimensions and computation time for fabric image retrieval. Zhang et al.^31^ have presented aggregated convolutional descriptors and approximate nearest neighbours search approach to combine texture and colour features for wool fabric retrieval on a dataset of 82,073 wool images. Prasetyo and Akardihas^32^ used a CNN for retrieving Batik images on a small dataset, and Deng et al.^33^ proposed a focus ranking approach integrated with CNN for fine-grained fabric image retrieval. They produced a dataset of 25,000 fabric images from 4300 original images. Tena et al.^34^ proposed a Modified CNN model for a more accurate search of ikat woven fabrics on a dataset of 4800 images. Cui and Wong^35^ introduced a joint local PCA-based 2D color and 2D orientation feature descriptor for textile image retrieval, surpassing histogram features on a 1000 stripe, plaid, and pattern images dataset. Maji and Bose^36^ proposed a pre-clustering approach in CBIR using deep learning features on datasets like Caltech-101, Corel-1K, and DB2000 without using humans in the loop.

Limited efforts has been laid into visualizing the feature’s explainability in interactive CBIR. Rui et al.^37^ introduced the relevance feedback method for enhancing the retrieval process’s explainability. Imo et al.^38^ presented the visualization of color histograms and texture features to give the user an idea of what they have specified. The medical field has seen recent progress in this area^39–41^.

## Methodology

Pre-trained networks are models trained on large data sets and can be utilized as a starting point for specific tasks. They save time and resources as they can be adjusted for better accuracy and speed in computer vision and natural language processing. This paper represents a feature fusion approach for obtaining features from images by fusing the strengths of multiple pre-trained deep-learning models. Each model has learned distinctive features and representations from various data sets and tasks. By combining their abilities, we can tap into the unique information extracted by each network, resulting in a more comprehensive and distinct set of features. This approach allows us to capture a broader range of patterns and structures in the input data, thus enhancing the richness of our analysis.

To lay the foundation, we provide a concise overview of essential concepts like convolutional neural networks and pre-trained models in this section.

### Convolutional neural network

Convolutional neural network (ConvNet/CNN) is the frequently used deep learning algorithm. It can take input images, assign importance (learnable weights and biases) to aspects and objects in the image, and distinguish between them. The layers of CNN have neurons arranged in 3 dimensions: height, depth and width. The word depth implies the 3rd dimension of the layer’s activation volume. A layer’s neurons are linked to a small region of the preceding CNN layer rather than all of them, unlike in a completely linked neural network. Thus, a CNN comprises multiple layers, and each layer transforms activation volume from one to another via differentiable functions. Their essential components [(convolution, pooling, fully connected layer, and some activation layers (e.g., ReLU, softmax, etc.)] operate on local input regions and depend only on relative spatial coordinates, which is impossible with conventional neural networks. CNNs are recognized for their weight-sharing and local connectivity characteristics^11^. These two characteristics permit the CNNs to act like local filters and to detect the same pattern in more than one part of the image with lesser trainable parameters, reduce the model’s memory requirement, and improve the model’s statistical efficiency.

### Pre-trained neural network model

“Pre-training” refers to training models on a big benchmark dataset or task, preserving the trained weights or parameters as outputs. As a result, it reduces the number of steps needed for the model’s output to converge. It involves training a model or parameters on one dataset or task and then applying them to train another model on a different dataset or task. Pre-trained weights significantly reduce training time and improve efficiency despite starting with random weights. It gives the model a head start instead of beginning from scratch. Due to the substantial computational resources required to train deep learning models, importing and utilizing such models is a common practice. Canziani et al.^42^ conducted a comprehensive performance analysis of pre-trained models using ImageNet data in computer vision applications. Transfer learning, a widely used application of pre-trained models in computer vision^43,44^, leverages prior learning through these weights, leading to substantial time savings compared to starting training from scratch. Moreover, it often yields significantly better results. This study’s motivation lies in using the transferred knowledge, represented by the layer weights of pre-trained CNN models, as feature extractors. All convolution and pooling layers are frozen, requiring no further training. To determine the output class or value, fully connected (F.C.) layers are removed, and softmax classifier layers are added above these features. Fine-tuning the F.C. layers means utilizing these layers and the knowledge learnt from the source domain dataset (ImageNet) to fit to the target domain dataset (TIAD). As a result, the F.C. layers serve as the classifier, initialized with pre-trained weights. Thereby, it expedites training and facilitates quicker convergence.

### Proposed method used a deep feature fusion for feature extraction

The model architecture starts learning high-level (abstract) features from low-level features as it becomes more intense. To represent images in a CBIR system, we use higher-level features fused from multiple models. The working of retrieval systems depends immensely on the quality and discriminative power of the features extracted from images. Our research targets to retrieve images correctly. For this, we merge information from multiple models to boost retrieval accuracy. In addressing our research questions, we design a fused deep learning approach to automatically retrieve images in our proposed CBIR system, leveraging the strengths of pre-trained CNN models—InceptionResNetV2 and InceptionV3.

**Figure 2:**
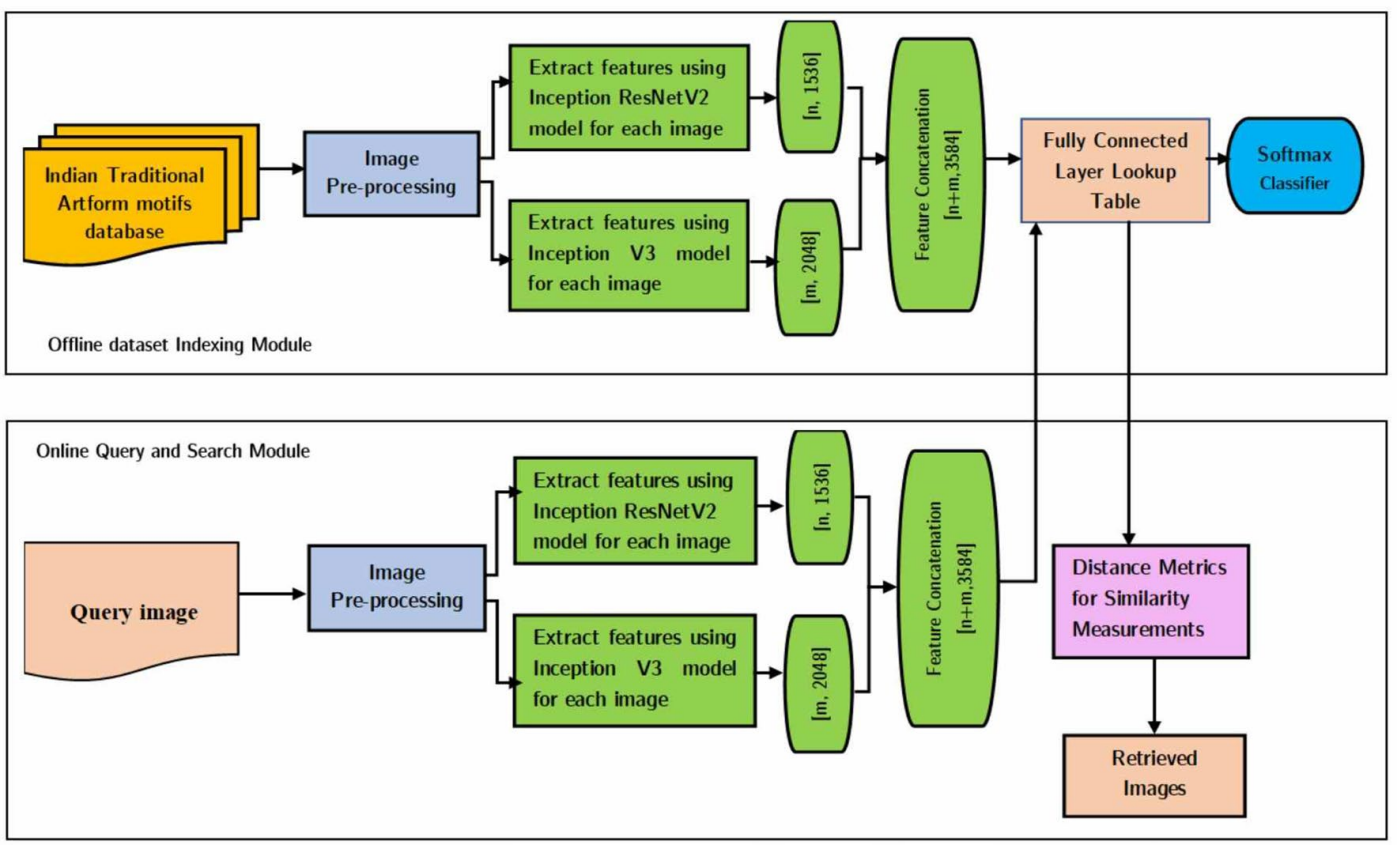
CBIR image feature presentation with the help of deep feature fusion between pre-trained learning models.

Our selection of InceptionResNetV2 and InceptionV3 as foundational CNN models for our CBIR architecture is a crucial starting point. This choice is not arbitrary; it stems from prior research and experiments explained in the subsequent section that have demonstrated their effectiveness. By concatenating the features from both networks, we achieve enhanced model performance, improved representation capabilities, robustness, and the ability to leverage distinct viewpoints for better understanding and generalization. This fusion produces a complementary feature representation, which can boost the overall model performance. Moreover, it helps minimize biases and limitations intrinsic to a single network architecture, resulting in more discriminative features and improved accuracy and generalization ability. We employ the concatenation method for feature fusion, directly merging the features from the networks. Each image’s resulting feature vector has a sum of the dimensions of the fused features. We discard the softmax activation layer to ensure the most informative representation and select the preceding fully connected layer as our feature vector for CBIR. This vector takes the learned high-level features of the models. We encode the images in the CBIR database by employing a pre-trained model higher level features fusion and obtain an (n+m)-dimensional feature vector for each image. The value of n and m varies with the deep learning network architecture selection. Here, the output features are generated from the InceptionResNetV2 and InceptionV3 network models with dimensions of 1536 and 2048, respectively. The benefit of this method is that it extracts higher-level features without relying on class information from our database. To avoid the laborious task of manually classifying images in our dataset, we use a pre-trained neural network model, which is trained on an independent dataset (ImageNet) for feature extraction. Figure 2 illustrates the flowchart of our feature fusion process.

## Details of the proposed algorithm and performance evaluation

### Dataset description

This dataset is an extended version of an earlier work^17,18^. Significant efforts have been laid into enhancing the size of datasets of Indian traditional art forms and their subclasses by gathering information from various connoisseurs at Taj Mahotsav (Agra), Delhi Haat (Delhi), and from websites such as FABCURATE, Matkatus, Pinterest-India, Sanskruti Yards of Tradition, Mandir, DEEPAM, and iMithila, etc.

The Traditional Indian Art Forms Dataset (TIAD) consists of 22,547 total images of nine styles, including Bagh (2570 images), Bandhani (2668 images), Batik (3078 images), Chikankari (2307 images), Ikat (2724 images), Kalamkari (1502 images), Kashida (2228 images), Madhubani (2280 images), and Warli (3190 images). The JPEG images are saved in 300 $\times$ 300-pixel resolution.

To further confirm the outcome of the recommended approaches, experiments are also performed on standard datasets available in the literature. Publicly available benchmark CBIR datasets taken in this work are as follows.

- Corel-1K: It contains 1000 images categorized into 10 categories containing 100 images each^45^.
- Caltech-101: It contains 9144 images classified into 101 categories. There are 34–800 images in each category^46^.

### Similarity measures

Once the feature vectors for total images in the database are computed and normalized, the task is to find the relevance of each image in the database to a provided query image. The most pertinent images are then retrieved as the final query result. The similarity (or dissimilarity) between a query image (Q) provided by the user and an existing image from the system database is measured by some distance metric. In this section, the following lists the similarity or dissimilarity measures we considered in our research. Let Q denote the vector ($Q_{1}$, $Q_{2}$, ..., $Q_{n}$) representing the query image and R the vector ($R_{1}$, $R_{2}$, ..., $R_{n}$) representing another image. Further, let $\bar{Q}$ represent the mean of the values in the Q vector and $\bar{R}$ the mean of R. Further, let q and r represent, respectively, the cumulative distributions of Q and R when they are considered as probability distributions ($\sum _{i=1}^{n}Q_{i}=\sum _{i=1}^{n}R_{i}=1$). That is Q = ($q_{1}, q_{2}, \cdots , q_{n}$) where $q_{j}$ = $\sum _{i=1}^{j}Q_{i}$ and similarly for r and R. Here, n is the feature dimension of the images and i is the *i*th feature value of the database and query image. Finally $\mu = (\mu _{1},\ldots ,\mu _{n})$ is the mean vector such that $\mu =\frac{Q+R}{2}$.

- Standard measures
  Euclidean distance ($$L_{2}$$)             
  1 $$\begin{aligned} d(Q,R) = \sqrt{\mathop \sum \limits _{i=1}^{n}(Q_{i}-R_{i})^{2}} \end{aligned}$$
  Cityblock distance ($$L_{1}$$)             
  2 $$\begin{aligned} d(Q,R)= \mathop \sum \limits _{i=1}^{n}|Q_{i}-R_{i}| \end{aligned}$$
- Divergence measures
  Jeffrey divergence (J.F.) (Puzicha et al.^47^)
  3 $$d(Q,R) = \sum\limits_{{i = 1}}^{n} {Q_{i} \log \frac{{Q_{i} }}{{\mu _{i} }} + R_{i} \log \frac{{R_{i} }}{{\mu _{i} }}} .$$
- Other measures
  Tanimoto coefficient (T.C.)             
  4 $$\begin{aligned} d(Q,R)=\frac{Q.R}{{||Q||^2}+||R||^2-{Q.R}} \end{aligned}$$

### Evaluation of performance

We calculate the performance of the CBIR system using precision and recall as a measure, which is defined as follows.

5 $$\begin{aligned} \begin{aligned} Precision&= \frac{ \Bigl (Number\ of\ true\ positives\Bigr )}{\Bigl (Number\ of\ true\ positives\ + Number\ of\ false\ positives\Bigr )}&= \frac{\Bigl (Number\ of\ relevant\ images\ retrieved \Bigr )}{\Bigl (Number\ of\ retrieved\ images \Bigr )} \end{aligned} \end{aligned}$$

Precision is the ratio of true positives to the total number of retrieved images. It represents the accuracy of the CBIR system in retrieving relevant images. Normally, the number of images retrieved by any CBIR method is a pre-specified positive integer. It is termed as the scope of the system. Precision value is computed for each image in the database, and these values are averaged over all images. Usually, the greater the scope, the more significant the number of relevant images retrieved, leading to decreased Precision.

6 $$\begin{aligned} \begin{aligned} Recall&= \frac{ \Bigl (Number\ of\ true\ positives\Bigr )}{\Bigl (Number\ of\ true\ positives\ + Number\ of\ false\ negatives\Bigr )}&= \frac{\Bigl (Number\ of\ relevant\ images\ retrieved \Bigr )}{\Bigl (Total\ Number\ of\ relevant\ images\ in \ the\ dataset \Bigr )} \end{aligned} \end{aligned}$$

Recall is another performance measure in CBIR systems that evaluates the ability of the system to retrieve relevant images from a given query. It represents the ratio of relevant images retrieved to the database’s total number of relevant images. Higher recall values indicate better system performance in retrieving relevant images.

## Results

This section describes the choice of the preeminent pre-trained models employed for fusion, the selection of the most effective similarity measure for our fusion architecture utilizing deep learning network features, the retrieval results of our CBIR system for the selected query images extracted from our datasets, and the precision and recall of image retrieval organized by categories within our dataset.

### Model selection

We have experimented with various pre-trained model architectures and found that InceptionResNetV2 and InceptionV3 models perform exceptionally well on our TIAD dataset, shown in Table 1. This approach improves the accuracy efficiency and saves time for image retrieval in our database.

**Table 1:** Comparison between pre-trained model’s performance for a scope value 20 on the TIAD dataset, using Euclidean distance for similarity measure.

Model name	Retrieval average precision (%)
InceptionResNetV2^48^	88.65
InceptionV3^48^	88.24
VGG19^49^	84.00
VGG16^49^	83.00
Xception^50^	87.00

### Selection of best similarity measure

Various types of distance measures are employed to determine the similarity or dissimilarity between images in the CBIR system. We took 1500 random images from each class, applied these images one by one, and retrieved the top 20 images. Then, determined the average precision for every class. The results shown in Table 2 show that the Manhattan City block distance measure is the winner with a 92.46% average precision value, the best-retrieved category is Kalamkari (precision: 95.0%), and the worst category is Chikankari (precision: 89.12%). The results showed that Manhattan City block distance and Tanimoto coefficient distance measure provided better results than Euclidean and Jeffrey distance measures. Therefore, we use the Manhattan City block distance measure for all the succeeding experiments.

**Table 2:** Comparison between various distance metrics on TIAD dataset.

IRV2+IV3				
Methods	Euclidean	Manhattan City block	Jeffrey	Tanimoto coefficient
Database classes	Average precision	Average precision	Average precision	Average precision
BAGH	0.90202	**0.93242**	0.90164	0.92020
BANDHEJ	0.91599	**0.92299**	0.91599	0.91846
BATIK	0.91414	0.92020	0.91000	**0.92534**
CHIKANKARI	0.87399	**0.89124**	0.86443	0.88442
IKAT	0.90576	**0.92046**	0.905769	0.91000
KALAMKARI	0.93958	**0.95000**	0.93862	0.94122
KASHIDA	0.92043	0.92365	0.92043	**0.92412**
MADHUBANI	0.92358	**0.93915**	0.90232	0.92358
WARLI	0.91683	**0.92168**	0.89886	0.91683
Overall average	0.91248	**0.92464**	0.90645	0.91824

The highest value among the distance measures of each class are in bold.

### Sample query image retrieval

**Figure 3:**
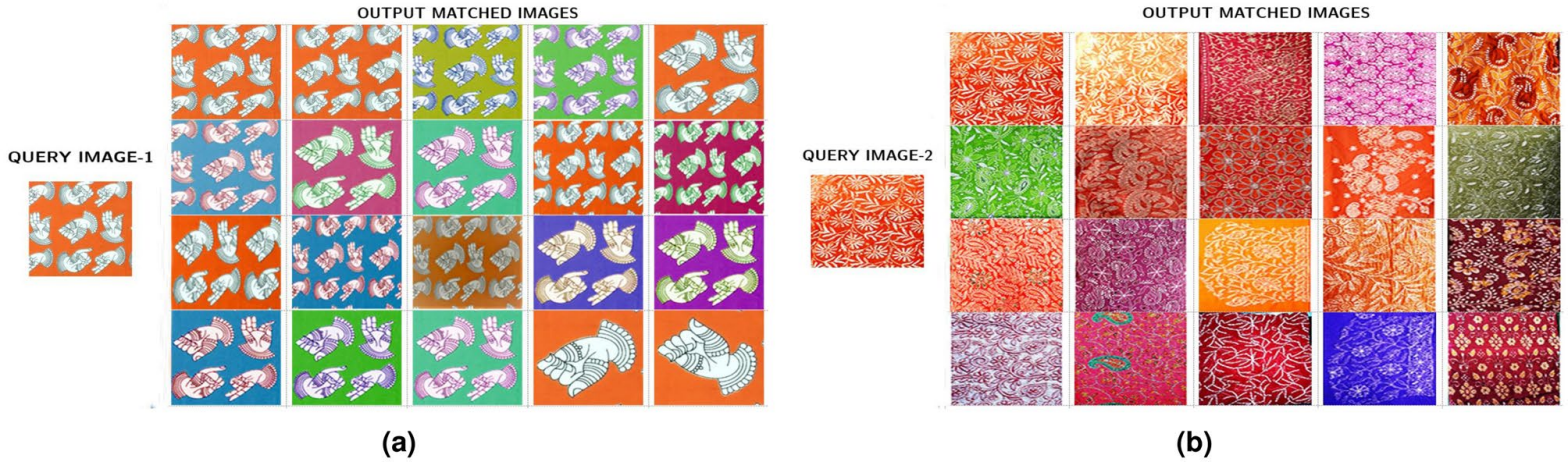
Output matched images (**a**) from the query image-1, and (**b**) query image-2.

For the scope of 20, using the deep feature fusion architecture on the TIAD Dataset, the sample query image retrieves 20 results as depicted in Fig. 3a. Human experts have manually evaluated and annotated these images based on the defined relevance criteria that serve as our ground truth.

From retrieval results, we find that the query images are from the “kalamkari” category, and all 20 results are related to the query image. Hence, the precision for this query image is 1. The total number of relevant images in the dataset is 40, so the recall for this query image is 20/40 = 0.5.

For one more query image from the TIAD dataset, the retrieved results are depicted in Fig. 3b. Here, we observe that the query image falls in the “Chikankari” category; out of 20 retrieved results, 16 are related to the query image. The total number of relevant images in the dataset is 60. Hence, for this specific image, the precision value is 16/20 = 0.80,and the recall value is 16/50 = 0.32.

### Class-wise average precision and recall calculation

**Table 3:** Class-wise average precision and recall for a scope of 20 on (a) TIAD, and (b) Corel-1K.

Class	Precision (%)	Recall (%)
(a) TIAD dataset		
Bagh	93.24	23.13
Bandhej	92.29	17.95
Batik	92.02	15.12
Chikankari	89.12	18.00
Ikat	92.04	17.24
Kalamkari	95.00	26.46
Kashida	92.36	16.11
Madhubani	93.91	24.26
Warli	92.17	17.33
Mean	92.46	19.51
(b) Corel-1K dataset		
African People	83.65	18.60
Beach	97.26	20.05
Building	95.00	20.43
Bus	100.00	20.00
Dinosaurs	100.00	20.00
Elephant	100.00	20.00
Flower	98.50	19.12
Food	96.05	19.12
Horse	100.00	19.80
Mountain	99.00	20.84
Mean	96.99	19.79

In this subsection, we generate the class-wise average precision and recall on TIAD, Corel-1K dataset for a scope of 20 using Manhattan City-block Distance as a similarity metric. Table 3a shows that for the Kalamkari class in the TIAD dataset, the proposed fusion architecture retrieves the highest 95.00% precision and 26.46% recall. However, the Chikankari class reflects the lowest precision, 89.12%, and the Batik class retrieves the lowest recall 15.12%. The performance of the Kalamkari class indicates that the proposed architecture is very effective at accurately identifying and retrieving images relevant to that class. The overall mean precision for this dataset is 92.46%, and recall is 19.51%.

Table 3b shows that in the Corel-1K dataset, for Bus, Dinosaurs, Elephant, and Horse classes, the proposed fusion architecture retrieves the highest 100.00% precision, but for the African People class performs the lowest 83.65% precision. The highest average recall value for the Mountain class is 20.84%, and the lowest is 18.60% for the African People class. This dataset’s overall mean precision and recall are 96.99% and 19.79%.

### Comparing results with other authors’ proposed algorithms

**Figure 4:**
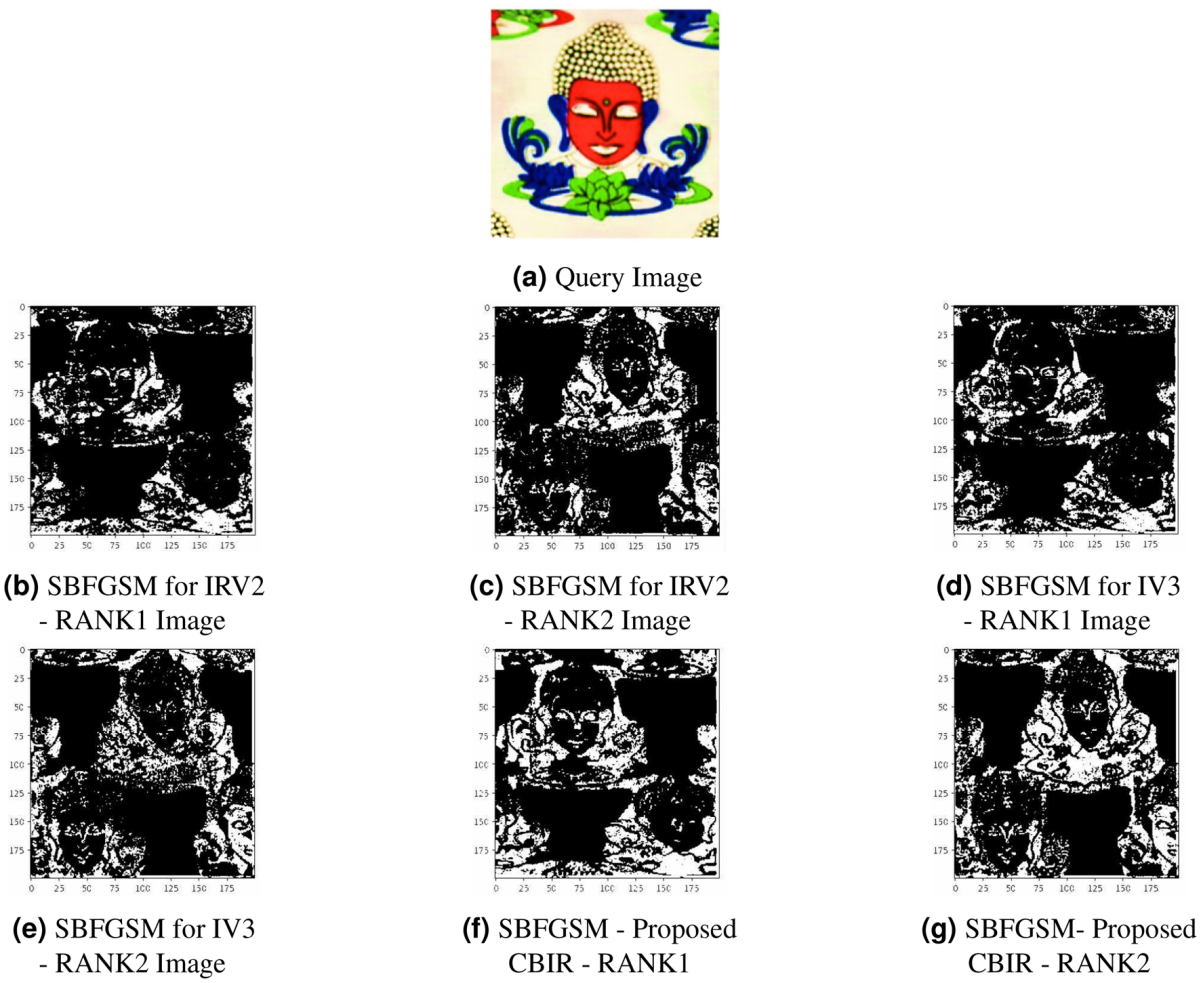
Visualization of features retrieval results using SBFGSM approach.

For dataset Corel-1K and Caltech-101, we select Maji and Bose^36^ paper as the baseline result. This paper^36^ extracted deep features from the images using InceptionResNetV2 CNN without using Relevance feedback. We are comparing the precision and recall of this paper^36^ with ours. Many works^36,51–60^ have been done on Corel-1K dataset, extracting various features and similarity distances. Figure 4a indicates that our recommended method is more accurate than the discussed methods in Maji and Bose^36^.

For the Caltech-101 Dataset, we took the average precision and recall of the finest methods applied in paper^36,53,54,61,62^. Results are depicted in Table 4b. The recommended method outclassed other methods.

## Simulated visualization using similarity-based fine-grained saliency maps

This section discusses the vital role of fusion features and introduces a new and advanced method called similarity-based fine-grained saliency maps (SBFGSM) in our innovative content-based image retrieval system. This unique technique visualizes the crucial features within an image and showcases remarkable superiority over individual models.

In this part, we discuss essential fusion features and introduce our new and advanced approach called similarity-based fine-grained saliency maps (SBFGSM) in our content-based image retrieval (CBIR) system. This technique helps us see the essential parts of an image and is much better than using separate models.

Fusion features play a critical role in addressing the limitations of single-model-based CBIR systems. Some key reasons why fusion features are crucial:

1. Enhanced discriminative power: fusion features integrate complementary information from different sources, thereby increasing the discriminative power of the retrieval system. By combining diverse aspects of image content, we can capture a broader range of visual cues and semantic information.
2. Robustness to variability: single models may exhibit limitations in handling variations in image content, such as lighting conditions, viewpoints, and occlusions. Fusion techniques help mitigate these limitations by aggregating information from multiple models, making the system more robust to diverse scenarios.
3. Improved retrieval accuracy: fusion features enable more effective matching between query and database images. By incorporating different modalities or representations, the retrieval system can better align with the user’s intent, improving accuracy in retrieving relevant images.

To demonstrate the advantages of fusion features, we utilize similarity-based fine-grained saliency maps (SBFGSM). This technique leverages the following principles:

1. Saliency-driven fusion: our method computes SBFGSM for each image, highlighting regions of interest based on their relevance to the query. By integrating these maps with the features from InceptionV3 and InceptionResNetV2, we create fusion features driven by the images’ visual saliency.
2. Enhanced retrieval relevance: the fusion features generated by our approach exhibit improved retrieval relevance compared to using individual models alone. The visual saliency maps guide the fusion process, emphasizing semantically significant regions, leading to more accurate retrieval results.

Our proposed similarity-based fine-grained saliency map (SBFGSM) approach can explain why a black-box CNN features a fused model (here, IRV2 and IV3 fusion), makes retrieval decisions by generating important region perturbation saliency maps for each decision^63–66^. A fine-grained saliency map refers to a detailed and localized representation of the most significant and visually distinct features within an image, allowing for precise analysis and understanding of specific regions or objects of interest and indicating how a particular region on the retrieved image impacts the similarity. However, a classification-based saliency map explains why a particular class label was assigned to an image, while a CBIR-based saliency map explains why specific images were considered similar to a query image during the retrieval process. Our SBFGSM measures how result regions contribute to the CBIR’s distance metric when computing similarity. In simple terms, the SBFGSM can be considered a heatmap in which brighter regions signify a higher contribution to the match score with the query, whereas darker areas have less impact.

We measure the the significance of a retrieved image region by applying a binary mask to block out the region of concern that perturbs it and observe how much this affects the black box decision. Inside the binary mask, the region of concern is 0; all other pixels have 1. We use a square block. By sliding the square block over the retrieval image by a stride step, we can show the blocked areas’ importance in impacting the similarity. Given a query image Q, a retrieval image R, $\odot$ denotes element-wise multiplication, $\mathbb {I}$ is a matrix with all entries are 1 and the same shape as $m_{i}$, $\parallel \overrightarrow{V_{1}},\overrightarrow{V_{2}} \parallel$ used L1Norm (Manhattan City Block distance) for the similarity between two vector and a square binary mask $m_{i} \in M$, the importance of the region-blocked out by $m_{i}$ is depicted as conveyed in Eq. (7):

7 $$\begin{aligned} K(Q,R,m_{i})= & {} max(( L1Norm(V_{R}\odot m_{i},V_{Q})-L1Norm(V_{Q}, V_{R})),0) \end{aligned}$$

8 $$\begin{aligned} SBFGSM(Q,R,M)= & {} \sum _{i}^{N} K(Q,R,m_{i})\odot \frac{1}{\sum _{i}^{N}(\mathbb {I}- m_{i})} \end{aligned}$$

In this, N is the top N retrieval image returned by the CBIR, and M is the set of the binary mask of the retrieved image with N binary masks. The succinct pseudo code of this approach is as follows.

**Figure:**
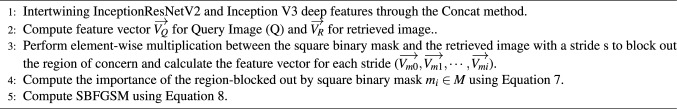
Algorithm 1.

Our objective of using the SBFGSM approach is to enhance the interpretability and visualization of the features extracted by feature fusion CNN architectures, thereby improving the transparency and user understanding of the CBIR system. We present the demonstration of the effectiveness of our fusion features, including the similarity-based fine-grained saliency maps, in Comparison to using InceptionV3 and InceptionResNetV2 as standalone models to showcase the superiority of our approach, shown in Fig. 4. It is clearly shown from Fig. 4 that more feature information is retrieved in our fusion approach, as visualized by our proposed SBFGSM approach, and brighter retrieved image information is obtained as compared to standalone models.

**Table 4:** Comparison between various authors’ proposed methods average precision and recall for a scope of 20 on (a) Corel-1K, and (b) Caltech-101 datasets.

Methods	Average precision (%)	Average recall (%)
(a) Corel-1K		
Proposed method	**96.99**	19.79
Maji and Bose^36^	96.11	19.65
Ghozzi et al.^51^	88.65	19.14
Singh and Batra^52^	92.00	18.4
Ahmed et al.^54^	76.50	13.60
Ahmed et al.^53^	83.50	12.30
Ashraf et al.^55^	73.05	14.50
Mehmood et al.^56^	87.85	17.37
Yousuf et al.^57^	85.20	17.00
Ahamed et al.^58^	82.00	19.00
Ashraf et al.^59^	82.00	16.40
Rashno et al.^60^	65.95	13.19
(b) Caltech-101 datasets		
Proposed method	**83.24**	19.37
Maji and Bose^36^	82.02	19.00
Ahmed et al.^54^	65.30	17.80
Ahmed et al.^53^	65.70	18.60
Rana et al.^61^	66.66	8.50
Bose et al.^62^	42.87	–

Our proposed approach gave the highest average precision value compared to other papers, as indicated in bold.

## Quick response CBIR system

Combining different types of pretrained models like InceptionResNetV2 and InceptionV3 has improved our results. However, we now need to see how quickly we can retrieve images, as we have a substantial dataset with many features. So this section, is about the pace of our CBIR system. We will indicate that it can find images for the TIAD and Caltech-101 datasets. We use principal component analysis^67^ and clustering to make this process faster without sacrificing accuracy. We are not calculating the time of image retrieval for Corel-1K because its dataset is small, and the result would not be meaningful.

### Principal component analysis (P.C.A.)

It is a dimensionality-reduction technique used to trim down many options into a limited subset that retains the bulk information in the primary data by lowering the number of possibilities. P.C.A. aims to decrease the information in a data set while retaining maximum information to the extent possible. A dimensionality decrease technique entails sacrificing some information for ease, as smaller data sets are easier to handle, visualize and quicker for machine learning algorithms.

### P.C.A. on concatenated deep features

A fused CNNs (IRV2 (extracted 1536 features) and IV3 (extracted 2048 features)) concatenated feature dimension is 3584, which is significant. So, we applied P.C.A. on the 3584 feature vector to lower its dimension and choose the number of primary components (M) with maximum average precision value. For the TIAD dataset, we are taking 1024 PCs, and for the Caltech-101 dataset, we are taking 100 PCs to resolve the precision. The first handful of P.C.s have approximately same or sometimes finer average precision for the 3584 features. For the TIAD dataset, the Average precision value with P.C.A. is 92.95%, and the average precision without P.C.A. is 92.46%. For the Caltech-101 dataset, the Average precision value with P.C.A. is 83.24%, and the average precision without P.C.A. is 83.24%.

In this approach, we attempted to analyze the time of average query image retrieval for the top 20 images on TIAD and Caltech-101 datasets. To demonstrate the working of P.C.A. employed in this work, we first train all database images through the CBIR fusion model (without the last softmax layer) and P.C.A., respectively. After that, we store these extracted features of dimension 1024 for TIAD and 100 for the Caltech-101 dataset of each image in memory as a feature bank. When a query image appears, it goes through the CBIR fusion model and P.C.A., respectively. Then, the features extracted from the query image are matched with each feature list in the feature bank. It ultimately retrieves those images with features closest to the query image features evaluated by Manhattan Distance. So, the time between supplying the query image followed by retrieval of similar images is termed image retrieval time. Table 5 shows this average image retrieval time. This can be clearly seen in Table 5 that using P.C.A. has lowered the retrieval time a bit.

**Table 5:** Compare the retrieval time with/without P.C.A. on (a) TIAD, and (b) Caltech-101 datasets.

Methods	Average retrieval time (in seconds)
(a) TIAD	
**With P.C.A**	**0.6586**
Without P.C.A	0.7435
(b) Caltech-101	
**With P.C.A**	**0.5837**
Without P.C.A	0.6874

The smallest average retrieval time value among the methods compared are in bold.

### Indian style Navratan clustering approach

Based on our previous discussion, image retrieval times increase as database size increases. We present an approach for speeding up the retrieval of images to address this issue. Our approach involves clustering images in the database and searching for images within specific clusters. Make a cluster of individual classes because each class has a unique texture to distinguish it from others like chikankari uses white color threads in its style. In contrast, Kalamkari contains natural color block printing and a pen for creating designs. To reduce model confusion, we only reduce the feature space to a specific class. In this study, we are using nine unique traditional styles popular worldwide. That’s why we name it “NAVRATTAN STYLE CLUSTERING”. To implement this method, we utilized the pre-trained CNN models InceptionResNetV2 and InceptionV3. Initially, these models were trained on ImageNet dataset to predict 1000 classes. However, we are fusing these models’ last convolution layer features by selecting the last dense layer for feature extraction and removing the last softmax layer.

**Figure 5:**
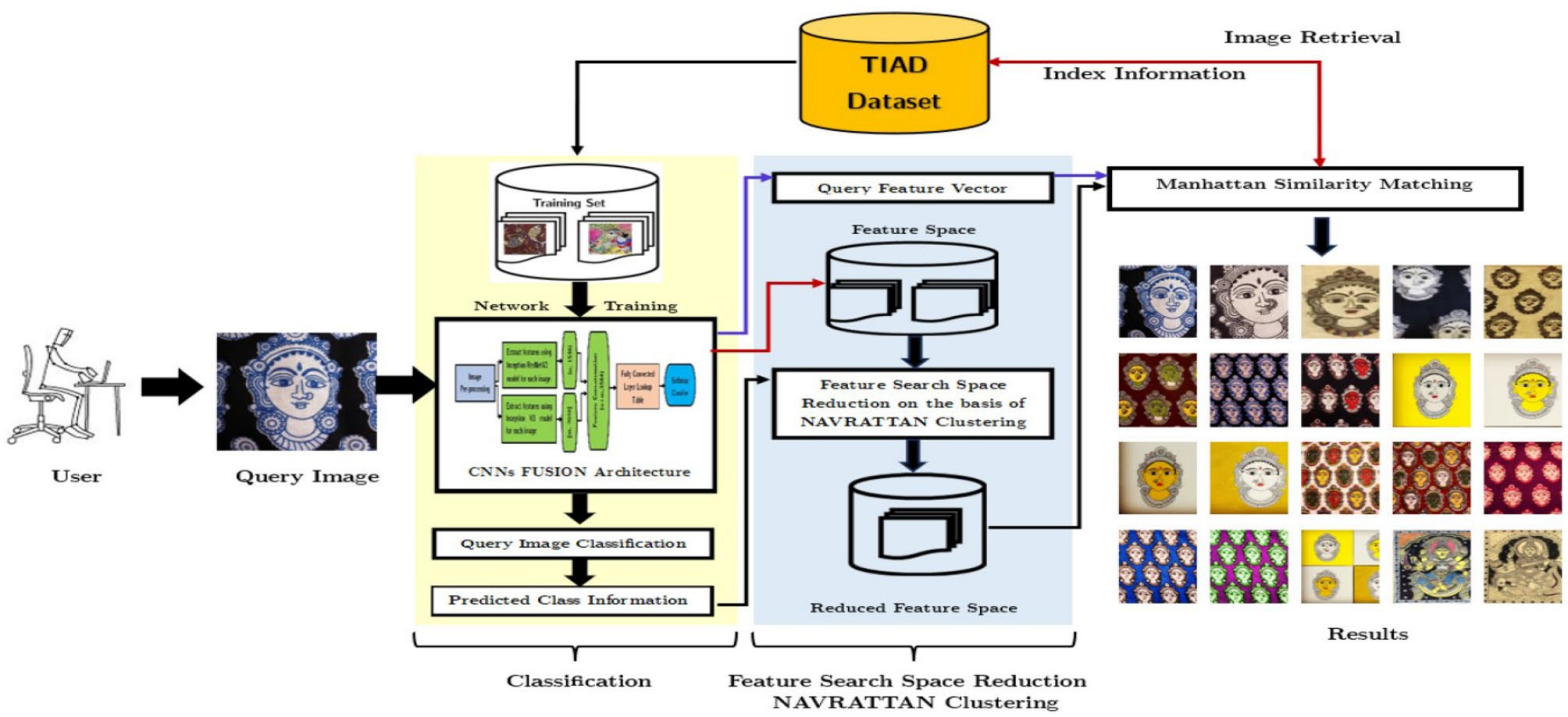
Schematic flow diagram of the recommended NAVRATTAN clustering approach for CBIR.

This method has been effectively implemented to the TIAD dataset. Figure 5 demonstrates the schematic flow diagram of the recommended NAVRATTAN STYLE clustering-based image retrieval.

The approach is illustrated below, step by step.

1. Firstly, we compute the last fully connected (fc) layer feature extraction, obtained from the concatenation of the last convolution layer features of both CNNs and train the fused model by transfer learning approach using 2048 neurons and 0.3 drop-out layer.
2. This newly trained fused model predicts the class $C_{q}$ of the query image $I_{q}$.
3. The final layer output of the trained fused model is utilized to extract the deep features of dataset images $Ij \in Z_{n}$ and the query image $I_{q}$.
4. The fused model is employed to construct a feature space $FS_{n}$, of image dataset $Z_{n}$. The image dataset contains n different images of 9 classes. It is represented as $Z_{n}$ = $\left\{ C_{1}\right\}$, $\left\{ C_{2}\right\}$, $\left\{ C_{3}\right\}$, $\ldots$,$\left\{ C_{9}\right\}$ where, $C_{i}$ represents the set of images, that allied to the *i*th class of $Z_{n}$. The feature space $FS_{n}$ is represented as $FS_{n}$=$\left\{ FS_{1}^{C}\right\}$, $\left\{ FS_{2}^{C}\right\}$, $\left\{ FS_{3}^{C}\right\}$, $\ldots$,$\left\{ FS_{9}^{C}\right\}$, where, $FS_{i}^{C}$ is the set of feature vectors of all images belonging to the *i*th class of $Z_{n}$.
5. In the next step, the predicted class information $C_{q}$ of query image $I_{q}$ is put to lower the feature space size $FS_{n}$.
6. To check the condition:
  if (query_class_label== prediction_value):
  then specific class cluster $(C_{q})$ selected for reduced feature space $(\hat{{FS}_{N}})$. This reduced feature space $(\hat{{F.S.}_{N}})$ contains deep feature vectors in the images resembles to the predicted class only. The reduced feature space $\hat{{FS}_{N}}$ contains feature vectors of $\forall I_{j} \in C_{q}$ where, $C_{q} \subset Z_{n}$. Thus, the lowered feature space $\hat{{FS}_{N}}$ is defined as: $\hat{{FS}_{N}}$ = $\left\{ FS_{q}^{C}\right\}$, where, $q \in {1, 2, 3, \cdots , 9}$. As a result, the lowered feature space $\hat{{FS}_{N}}$ contains drastically lesser feature vectors in contrast to the ${FS}_{N}$.
  Retrieve Top 20 identical images from $\hat{{FS}_{N}}$ using Manhattan city block distance measure.
  else
  No cluster be selected
  Retrieve Top 20 identical images from ${{FS}_{N}}$ using Manhattan city block distance measure.
7. As a result, the classification clustering drastically reduces the image search space based on the semantic nature of the clusters.
8. Retrieval time has been further reduced by applying the P.C.A. approach on reduced query vectors in 9 classes.
9. This approach saves little retrieval time, but it extracts more semantically analogous images in the retrieved output.

### Approach

**Figure 6:**
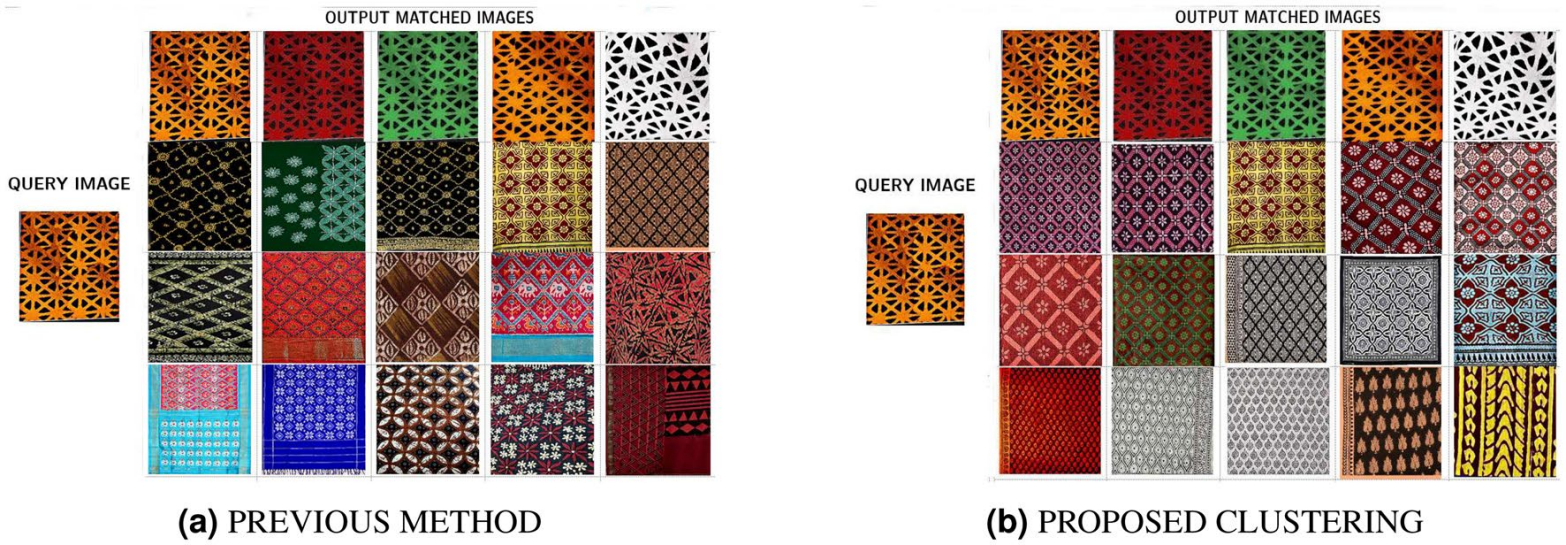
Proposed approaches retrieval outputs with scope 20.

The assessment of the outcome of this work is done on the TIAD dataset. Figure 6 depicts the retrieval outputs in comparison between the proposed clustering and the previous method for the “Bagh” category query image. Human experts have manually evaluated and annotated these images in reference to the defined relevance criteria that serve as our ground truth. It is clearly evident that the suggested clustering extracts more semantically identical images in the retrieved output than the previous approach. The retrieval efficiency for this specific query in the prior approach is 8/20 = 0.40, whereas for the clustering approach, it is 1, displayed in Fig. 6a,b.

Table 6 shows the faster retrieval time. This work has been inspected by both ways, i.e. with P.C.A and without P.C.A. From the time we feed the query to the system until we get the retrieved images is the retrieval time of an image. The process is kept repeated, treating every database images as query images. We noted down the retrieval time for every images and finally took the mean to determine the mean image retrieval time. Precision for the NAVRATTAN clustering retrieval method is 95.18%, improved from the earlier method’s precision of 92.46%. However, decrease in image retrieval time is significant, almost 1.47 times faster. The fused model predicts identical images within the similar classes, forming clusters of identical images. Therefore, we still obtain enhanced results even when searching within a smaller subset.

**Table 6:** Comparison between proposed approaches image retrieval time on TIAD dataset (a) with P.C.A, and (b) without P.C.A.

Proposed approaches	Average retrieval time (in seconds)
(a) With P.C.A	
**Navrattan clustering**	**0.4475**
Previous approach	0.6586
(b) Without P.C.A	
**Navrattan clustering**	**0.5494**
Previous approach	0.7435

The smallest average retrieval time value among the approaches compared are in bold.

### Relevance feedback using Manhattan City block distance measure

The relevance feedback (R.F.) concept originated from documentary information retrieval^68,69^. It has gotten much attention in the CBIR field, e.g., (Zhou and Huang^70^), since past few years. A relevance feedback mechanism is an additional tool for lowering the angle between user relevance and system relevance by giving a clearer vision of the user expectations and adjusting the inside system behavior to bridge the semantic gap. In our research, we attempted the Manhattan City block distance matching measure for ranked images displayed to a user. The user can record his feedback by marking interesting images as relevant, and the remaining images are inevitably considered irrelevant. This process is carried out for some iterations and stops when the user is convinced with the displayed results. The succinct pseudo code of this approach is as follows.

**Figure:**
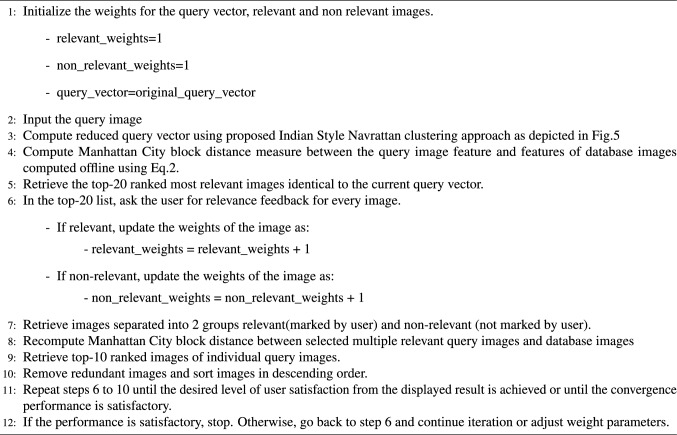
Algorithm 2: Proposed relevanace feedback approach.

### Approach

The CBIR system suggested by Maji and Bose^36^, which uses InceptionResNetV2 (IRV2) features, is without relevance feedback. We have attempted IRV2 and IV3 features with relevance feedback on our TIAD dataset and two publicly available datasets as a baseline. After that, we compared this with our proposed NAVRATTAN clustering approach. The precision measure is employed to evaluate retrieval performance, also termed as retrieval efficiency. Table 7 depicts the steady increase in retrieval efficiency of the TIAD dataset over 4 R.F. iterations.

**Table 7:** Proposed relevance feedback approach retrieval efficiency performance on the TIAD dataset.

Features	Method	Distance	RF iteration number				
			0 (%)	1 (%)	2 (%)	3 (%)	4 (%)
IV3	Baseline	Manhattan	89.89	92.03	94.67	95.14	95.96
IRV2	Baseline	Manhattan	90.30	92.17	95.03	95.89	96.24
**IRV2 + IV3**	Proposed	Manhattan	95.18	97.07	98.03	98.66	**99.00**

The highest average retrieval efficiency value among the methods compared are in bold.

Tables 8 provide details of the regular increase in retrieval performance over 3 R.F. iterations on the 2 databases listed as above, using the baseline and the proposed methods with the Manhattan distance.

**Table 8:** Proposed relevance feedback approach retrieval efficiency performance on the Corel-1K, and Caltech-101 datasets.

Dataset	Features	Method	Distance	RF Iteration number			
				0 (%)	1 (%)	2 (%)	3 (%)
Corel-1K	IV3	Baseline	Manhattan	94.50	95.67	96.43	97.15
	IRV2	Baseline	Manhattan	96.11	97.15	97.93	98.04
	**IRV2+IV3**	Proposed	Manhattan	98.09	98.98	99.75	**100.00**
Caltech-101	IV3	Baseline	Manhattan	81.33	84.17	87.90	90.05
	IRV2	Baseline	Manhattan	82.02	87.32	90.04	91.56
	**IRV2+IV3**	Proposed	Manhattan	87.24	89.75	92.51	**94.13**

The highest average retrieval efficiency value among the methods compared are in bold.

**Figure 7:**
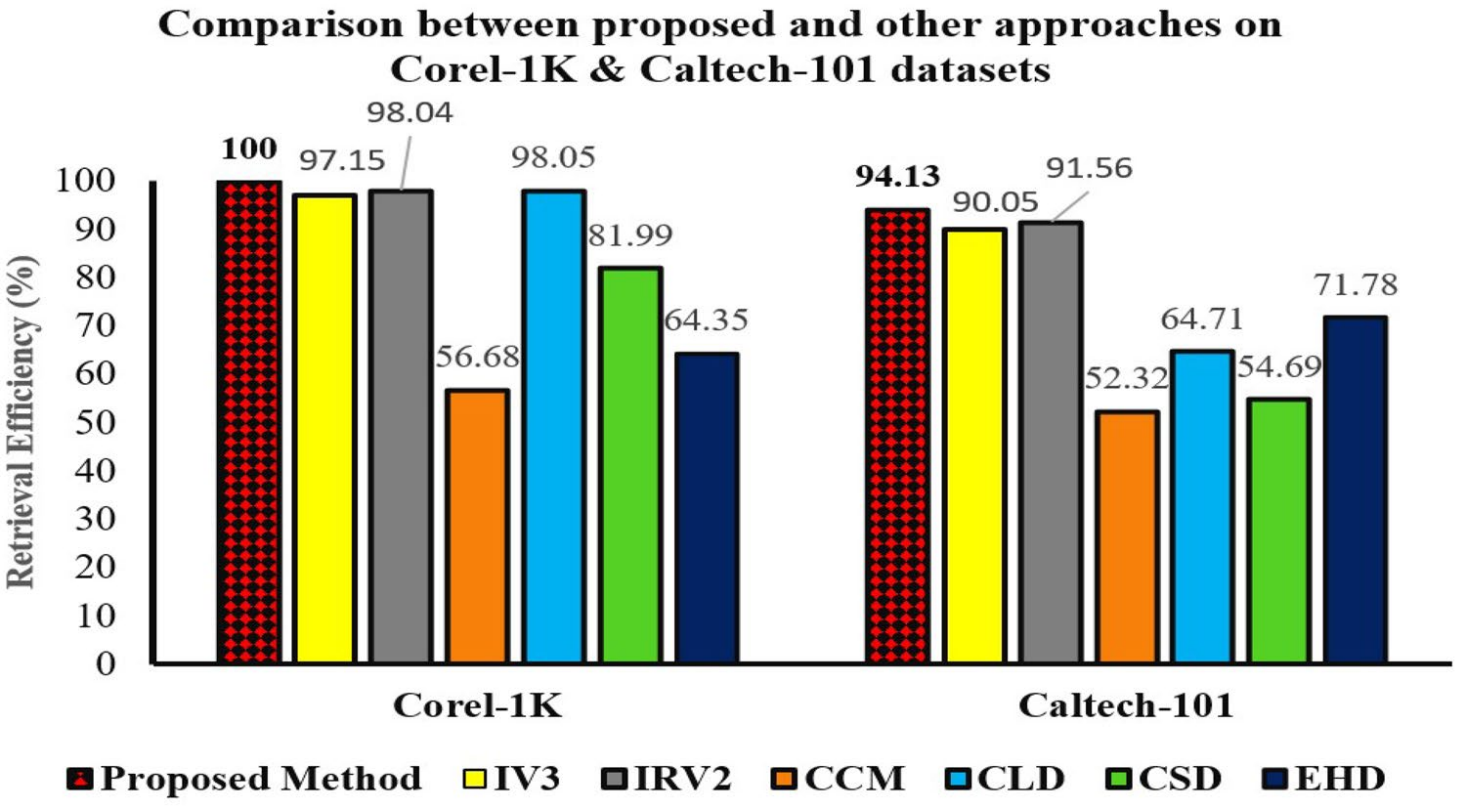
Comparison of CBIR methods performance on recommended approach and other methods.

Figure 7 and exhibits that our recommended method betters the methods discussed in Maji and Bose^36^ on two publicly available datasets. The retrieval efficiency corresponding to the most notable retrieval performance for each database is highlighted in bold in the respective figure.

## Conclusion

Using CBIR in Indian traditional textile motifs is crucial for preserving and promoting the rich background and cultural significance of Indian art forms. CBIR allows for the precise analysis and identification of intricate and detailed motifs, which is essential in Indian art form design.

We have created an expanded dataset of Indian Traditional Art forms, and our proposed approach of interactive CBIR is tested on this expanded dataset as well as on standard benchmark datasets. This study determines that utilizing a pre-trained fused model’s last layer features returns more precise “precision results” and “recall results” for CBIR than traditional methods such as C.C.M. and wavelet. The similarity-based fine-grained saliency maps (SBFGSM) algorithm has been proposed to display the significance of fusion features compared to single model features.

Additionally, we integrate several strategies to optimize CBIR retrieval efficiency and speed, in which the relevance feedback plays an important role. It facilitates more effective retrieval scores by permitting the user to provide feedback. This transparency empowers them by fostering user-friendly conditions for selecting relevant images in CBIR. With the help of Navrattan clustering, our interactive CBIR system has also tested on dataset image space and reducing features. This technique reduces retrieval time and efficiency in Indian art form design. Our proposed methods have broader applicability for their employability in other datasets and models to examine similar issues using saliency maps for image similarity analysis.

We are working on retrieving the same images with the same query image but with different orientation angles. Different datasets may have varying characteristics, such as image resolution and quality. The effectiveness of the content-based image retrieval (CBIR) methods proposed in the study could be influenced by these variations. In the future, we will introduce this feature in our interactive CBIR system.

## Data Availability

To obtain the datasets generated during the current study, interested parties may connect with the corresponding author and make a reasonable request.

## Code availability

Code will be made available on request, and interested parties may contact the corresponding author.

## References

[1] Lachkar A, Benslimane R, D’orazio L, Martuscelli E (2006). A system for textile design patterns retrieval. Part I: Design patterns extraction by adaptive and efficient color image segmentation method. J. Text. Inst..

[2] Birjandi M, Mohanna F (2020). 24 modified keyword based retrieval on fabric images. Quantum J. Eng. Sci. Technol..

[3] Gudivada VN, Raghavan VV (1995). Content based image retrieval systems. Computer.

[4] Müller H, Müller W, Squire DM, Marchand-Maillet S, Pun T (2001). Performance evaluation in content-based image retrieval: Overview and proposals. Pattern Recogn. Lett..

[5] Liu Y, Zhang D, Lu G, Ma W-Y (2007). A survey of content-based image retrieval with high-level semantics. Pattern Recogn..

[6] Tena S, Hartanto R, Ardiyanto I (2021). Content-based image retrieval for fabric images: A survey. Indones. J. Electr. Eng. Comput. Sci..

[7] Niblack, C. W. *et al.* Qbic project: Querying images by content, using color, texture, and shape. In *Storage and Retrieval for Image and Video Databases*, Vol. 1908, 173–187. 10.1117/12.143648 (Spie, 1993).

[8] Min R, Cheng H-D (2009). Effective image retrieval using dominant color descriptor and fuzzy support vector machine. Pattern Recogn..

[9] Zhang J, Ye L (2010). Series feature aggregation for content-based image retrieval. Comput. Electr. Eng..

[10] Santini S, Jain R (1999). Similarity measures. IEEE Trans. Pattern Anal. Mach. Intell..

[11] Krizhevsky A, Sutskever I, Hinton GE (2017). Imagenet classification with deep convolutional neural networks. Commun. ACM.

[12] Deng, J. *et al.* Imagenet: A large-scale hierarchical image database. In *2009 IEEE Conference on Computer Vision and Pattern Recognition*, 248–255. 10.1109/CVPR.2009.5206848 (IEEE, 2009).

[13] Russakovsky O (2015). Imagenet large scale visual recognition challenge. Int. J. Comput. Vision.

[14] Babenko, A., Slesarev, A., Chigorin, A. & Lempitsky, V. Neural codes for image retrieval. In *Computer Vision–ECCV 2014: 13th European Conference, Zurich, Switzerland, September 6–12, 2014, Proceedings, Part I 13*, 584–599. 10.1007/978-3-319-10590-1_38 (Springer, 2014).

[15] Li J, Allinson N, Tao D, Li X (2006). Multitraining support vector machine for image retrieval. IEEE Trans. Image Process..

[16] Zhou XS, Huang TS (2003). Relevance feedback in image retrieval: A comprehensive review. Multimed. Syst..

[17] Varshney S, Lakshmi CV, Patvardhan C (2023). Madhubani art classification using transfer learning with deep feature fusion and decision fusion based techniques. Eng. Appl. Artif. Intell..

[18] Varshney, S., Vasantha Lakshmi, C. & Patvardhan, C. Traditional Indian textile designs classification using transfer learning. In *Machine Learning, Image Processing, Network Security and Data Sciences: Select Proceedings of 3rd International Conference on MIND 2021*, 371–385. 10.1007/978-981-19-5868-7_28 (Springer, 2023).

[19] Arora, C., Vijayarajan, V. & Padmapriya, R. Content-based image retrieval for textile dataset and classification of fabric type using svm. In *Frontiers in Intelligent Computing: Theory and Applications: Proceedings of the 7th International Conference on FICTA (2018), Volume 2*, 304–314 (Springer, 2020).

[20] Xiang J, Zhang N, Pan R, Gao W (2022). Patterned fabric image retrieval using relevant feedback via geometric similarity. Text. Res. J..

[21] Jing J, Li Q, Li P, Zhang H, Zhang L (2015). Patterned fabric image retrieval using color and space features. J. Fiber Bioeng. Inform..

[22] Suciati, N., Herumurti, D. & Wijaya, A. Y. Fractal-based texture and hsv color features for fabric image retrieval. In *2015 IEEE International Conference on Control System, Computing and Engineering (ICCSCE)*, 178–182 (IEEE, 2015).

[23] Jing J, Li Q, Li P, Zhang L (2016). A new method of printed fabric image retrieval based on color moments and gist feature description. Text. Res. J..

[24] Nurhaida I, Wei H, Zen RA, Manurung R, Arymurthy AM (2016). Texture fusion for batik motif retrieval system. Int. J. Electr. Comput. Eng..

[25] Mutia C, Arnia F, Muharar R (2017). Improving the performance of CBIR on Islamic women apparels using normalized PHOG. Bull. Electr. Eng. Inform..

[26] Prasetyo H, Wiranto W, Winarno W (2018). Statistical modeling of gabor filtered magnitude for batik image retrieval. J. Telecommun. Electron. Comput. Eng..

[27] Yao L, Ke H (2019). Robust image retrieval for lacy and embroidered fabric. Text. Res. J..

[28] Xiang J, Zhang N, Pan R, Gao W (2019). Fabric image retrieval system using hierarchical search based on deep convolutional neural network. Ieee Access.

[29] Xiang J, Zhang N, Pan R, Gao W (2020). Fabric retrieval based on multi-task learning. IEEE Trans. Image Process..

[30] Sun J, Ding X-J, Du L, Li Q, Zou F (2019). Research progress of fabric image feature extraction and retrieval based on convolutional neural network. J. Text..

[31] Zhang N, Shamey R, Xiang J, Pan R, Gao W (2022). A novel image retrieval strategy based on transfer learning and hand-crafted features for wool fabric. Expert Syst. Appl..

[32] Prasetyo H, Akardihas BAP (2019). Batik image retrieval using convolutional neural network. Telecommun. Comput. Electron. Control (TELKOMNIKA ).

[33] Deng D (2018). Learning deep similarity models with focus ranking for fabric image retrieval. Image Vis. Comput..

[34] Tena S, Hartanto R, Ardiyanto I (2023). Content-based image retrieval for traditional Indonesian woven fabric images using a modified convolutional neural network method. J. Imaging.

[35] Cui Y, Wong WK (2018). Textile Image Retrieval Using Joint Local pca-Based Feature Descriptor. Applications of Computer Vision in Fashion and Textiles.

[36] Maji S, Bose S (2021). Cbir using features derived by deep learning. ACM/IMS Trans. Data Sci..

[37] Rui Y, Huang TS, Ortega M, Mehrotra S (1998). Relevance feedback: A power tool for interactive content-based image retrieval. IEEE Trans. Circ. Syst. Video Technol..

[38] Imo, J., Klenk, S. & Heidemann, G. Interactive feature visualization for image retrieval. In *2008 19th International Conference on Pattern Recognition*, 1–4. 10.1109/ICPR.2008.4761683 (IEEE, 2008).

[39] Ahmad J, Sajjad M, Mehmood I, Baik SW (2017). Sinc: Saliency-injected neural codes for representation and efficient retrieval of medical radiographs. PLoS One.

[40] Chittajallu, D. R. *et al.* Xai-cbir: Explainable AI system for content based retrieval of video frames from minimally invasive surgery videos. In *2019 IEEE 16th International Symposium on Biomedical Imaging (ISBI 2019)*, 66–69. 10.1109/ISBI.2019.8759428 (IEEE, 2019).

[41] Barata, C. & Santiago, C. Improving the explainability of skin cancer diagnosis using cbir. In *International Conference on Medical Image Computing and Computer-Assisted Intervention*, 550–559. 10.1007/978-3-030-87199-4_52 (Springer, 2021).

[42] Canziani, A., Paszke, A. & Culurciello, E. An analysis of deep neural network models for practical applications. arXiv:1605.07678 (arXiv preprint). 10.48550/arXiv.1605.07678 (2016).

[43] Torrey L, Shavlik J (2009). Transfer learning. Handbook of research on machine learning applications. IGI Glob..

[44] Marcelino, P. Transfer learning from pre-trained models (2019). https://towardsdatascience.com/transfer-learning-from-pre-trained-models-f2393f124751.

[45] Ortega-Binderberger, M. Corel image features data set. https://archive.ics.uci.edu/ml/datasets/corel+image+features. Accessed 23 Dec 2019. 10.24432/C5K599 (1999).

[46] Fei-Fei, L., Fergus, R. & Perona, P. Learning generative visual models from few training examples: An incremental Bayesian approach tested on 101 object categories. In *2004 Conference on Computer Vision and Pattern Recognition Workshop*, 178–178. 10.1109/CVPR.2004.383 (IEEE, 2004).

[47] Puzicha, J., Hofmann, T. & Buhmann, J. M. Non-parametric similarity measures for unsupervised texture segmentation and image retrieval. In *Proceedings of IEEE Computer Society Conference on Computer Vision and Pattern Recognition*, 267–272. 10.1109/CVPR.1997.609331 (IEEE, 1997).

[48] Szegedy, C., Ioffe, S., Vanhoucke, V. & Alemi, A. Inception-v4, inception-resnet and the impact of residual connections on learning. In *Proceedings of the AAAI Conference on Artificial Intelligence*, vol. 31. 10.1609/aaai.v31i1.11231 (2017).

[49] Simonyan, K. & Zisserman, A. Very deep convolutional networks for large-scale image recognition. arXiv:1409.1556 (arXiv preprint). 10.48550/arXiv.1409.1556 (2014).

[50] Chollet, F. Xception: Deep learning with depthwise separable convolutions. In *Proceedings of the IEEE conference on Computer Vision and Pattern Recognition*, 1251–1258. 10.48550/arXiv.1610.02357 (2017).

[51] Ghozzi Y, Baklouti N, Hagras H, Ayed MB, Alimi AM (2021). Interval type-2 beta fuzzy near sets approach to content-based image retrieval. IEEE Trans. Fuzzy Syst..

[52] Singh S, Batra S (2020). An efficient bi-layer content based image retrieval system. Multimed. Tools Appl..

[53] Ahmed KT, Ummesafi S, Iqbal A (2019). Content based image retrieval using image features information fusion. Inf. Fusion.

[54] Ahmed, K. T., Naqvi, S. A. H., Rehman, A. & Saba, T. Convolution, approximation and spatial information based object and color signatures for content based image retrieval. In *2019 International Conference on Computer and Information Sciences (ICCIS)*, 1–6. 10.1109/ICCISci.2019.8716437 (IEEE, 2019).

[55] Ashraf R (2018). Content based image retrieval by using color descriptor and discrete wavelet transform. J. Med. Syst..

[56] Mehmood Z, Mahmood T, Javid MA (2018). Content-based image retrieval and semantic automatic image annotation based on the weighted average of triangular histograms using support vector machine. Appl. Intell..

[57] Yousuf M (2018). A novel technique based on visual words fusion analysis of sparse features for effective content-based image retrieval. Math. Probl. Eng..

[58] Ahamed AMU, Eswaran C, Kannan R (2017). Cbir system based on prediction errors. J. Inf. Sci. Eng..

[59] Ashraf R, Bashir K, Irtaza A, Mahmood MT (2015). Content based image retrieval using embedded neural networks with bandletized regions. Entropy.

[60] Rashno, A. & Sadri, S. Content-based image retrieval with color and texture features in neutrosophic domain. In *2017 3rd International Conference on Pattern Recognition and Image Analysis (IPRIA)*, 50–55 (IEEE, 2017).

[61] Rana SP, Dey M, Siarry P (2019). Boosting content based image retrieval performance through integration of parametric and nonparametric approaches. J. Vis. Commun. Image Represent..

[62] Bose S, Pal A, Chakrabarti D, Mukherjee T (2017). Improved content-based image retrieval via discriminant analysis. Int. J. Mach. Learn. Comput..

[63] Fong, R. C. & Vedaldi, A. Interpretable explanations of black boxes by meaningful perturbation. In *Proceedings of the IEEE International Conference on Computer Vision*, 3429–3437. 10.1109/ICCV.2017.371 (2017).

[64] Ribeiro, M. T., Singh, S. & Guestrin, C. ”Why should i trust you?” Explaining the predictions of any classifier. In *Proceedings of the 22nd ACM SIGKDD International Conference on Knowledge Discovery and Data Mining*, 1135–1144. 10.1145/2939672.2939778 (2016).

[65] Zeiler, M. D. & Fergus, R. Visualizing and understanding convolutional networks. In *Computer Vision—ECCV 2014: 13th European Conference, Zurich, Switzerland, September 6–12, 2014, Proceedings, Part I 13*, 818–833. 10.1007/978-3-319-10590-1_53 (Springer, 2014).

[66] Dong, B., Collins, R. & Hoogs, A. Explainability for content-based image retrieval. In *CVPR Workshops*, 95–98 (2019).

[67] Shlens, J. A tutorial on principal component analysis. arXiv:1404.1100 (arXiv preprint). 10.48550/arXiv.1404.1100 (2014).

[68] Salton, G. Modern information retrieval. *(No Title)* (1983).

[69] Salton G (1989). Automatic Text Processing: The Transformation, Analysis, and Retrieval.

[70] Zhou XS, Huang TS (2002). Relevance feedback in content-based image retrieval: Some recent advances. Inf. Sci..

